# Remoteness influences access to sexual partners and drives patterns of viral sexually transmitted infection prevalence among nomadic pastoralists

**DOI:** 10.1371/journal.pone.0191168

**Published:** 2018-01-31

**Authors:** Ashley Hazel, James Holland Jones

**Affiliations:** 1 Department of Earth System Science, Stanford University, Stanford, California, United States of America; 2 Woods Institute for the Environment, Stanford University, Stanford, California, United States of America; 3 Division of Biological Sciences, Imperial College London, London, United Kingdom; Institut Catala De La Salut, SPAIN

## Abstract

Sexually transmitted infections (STIs) comprise a significant portion of the infectious-disease burden among rural people in the Global South. Particular characteristics of ruralness—low-density settlements and poor infrastructure—make healthcare provision difficult, and remoteness, typically a characteristic of ruralness, often compounds the difficultly. Remoteness may also accelerate STI transmission, particularly that of viral STIs, through formation of small, highly connected sexual networks through which pathogens can spread rapidly, especially when partner concurrency is broadly accepted. Herein, we explored the effect of remoteness on herpes simplex virus type-2 (HSV-2) epidemiology among semi-nomadic pastoralists in northwestern (Kaokoveld) Namibia, where, in 2009 we collected HSV-2-specific antibody status, demographic, sexual network, and travel data from 446 subjects (women = 213, men = 233) in a cross-sectional study design. HSV-2 prevalence was high overall in Kaokoveld (>35%), but was heterogeneously distributed across locally defined residential regions: some regions had significantly higher HSV-2 prevalence (39–48%) than others (21–33%). Using log-linear models, we asked the following questions: 1) Are sexual contacts among people in high HSV-2-prevalence regions more likely to be homophilous (i.e., from the same region) than those among people from low-prevalence regions? 2) Are high-prevalence regions more “functionally” remote, in that people from those regions are more likely to travel within their own region than outside, compared to people from other regions? We found that high-prevalence regions were more sexually homophilous than low-prevalence regions and that those regions also had higher rates of within-region travel than the other regions. These findings indicate that remoteness can create contact structures for accelerated STI transmission among people who are already disproportionately vulnerable to consequences of untreated STIs.

## Introduction

Rural communities suffer disproportionately from the global infectious disease burden [1]. While infectious disease is still the major cause of mortality in urban and rural sub-Saharan Africa [2, 3], the majority of the infectious diseases of poverty are suffered by the rural poor in developing countries [1]. Sexually transmitted infections (STIs) make up an important portion of this burden [4, 5], and complications from untreated non-HIV STIs are among the most significant causes of morbidity and mortality among rural women [6], indicating that STI treatment for these communities is neither adequate nor timely.

Delivering effective healthcare to rural areas is difficult for many reasons [7]. Rural, and particularly, remotely-living people are often far from healthcare facilities, which are concentrated in urban areas [8], making essential components of primary healthcare hard to access, leading to significant health disparities between rural- and urban-living people [9]. Contributing to the problem of access, remote communities are often sparsely populated. Healthcare improvements for small populations have modest impacts on public-health objectives, which de-incentivizes investing in improved access [10, 11]. Because of the particular implications remoteness has for human health, due to high subsistence mobility, indigeneity, and cultural isolation [12], it has been suggested that remoteness be considered as a distinct condition and not solely a secondary characteristic of rural living [13]. Furthermore, remoteness exacerbates the challenges presented by high-mobility livelihoods to identify infection “hot-spots,” estimate disease burden, provide treatment (including longterm management of incurable viral STIs), and conduct contact tracing and other infection-control measures, such as vaccination [14–16].

The health challenges facing rural communities are even greater among seemingly homogeneous cohesive populations, such as traditional indigenous communities with strong social rules and norms that may differ from those of the hegemonic population from which and for whom healthcare policies and services are usually designed. This may be partly due to lower understanding of and familiarity with clinical medicine. However, a critical and often overlooked factor may be environmental variations that underpin rural subsistence livelihoods, which could drive heterogeneities in disease risk. Subsistence-level societies are immediately dependent on their physical environments for their fundamental resource needs. Subsistence strategies are not the only part of human behavior that is shaped by local ecological conditions; many aspects of social behavior, including reproductive strategies, are influenced by opportunities for and constraints on resource access [17, 18].

Because of the important link between social strategies and ecological behavior, we argue that STI burden should be understood, at least in part, as an environmental health problem, particularly in remote subsistence communities. Despite the high variation among bacterial and viral STIs with regard to epidemic dynamics, both are a major source of disease burden in many remote sub-Saharan African communities [4]. In addition to the challenge of providing testing and treatment in remote settings that could reduce infection duration (curable bacterial infections), infectiousness (incurable viral infections), and incidence, concurrency of sexual partnerships is common, traditional, and strategically advantageous in remote populations with high subsistence mobility [19, 20]. Concurrency has been shown to increase the reproduction number (R_t_) of HIV, which, like herpes simplex virus type-2 (HSV-2), has a very low per-act transmission probability [21, 22]. Concurrency makes epidemics more likely and control more difficult.

Many STIs are simultaneously asymptomatic and infectious, owing to a variety of pathogen and host characteristics, including bacterial strain-specific immune evasion, viral latency, asymptomatic viral shedding, host immune status, and psychological normalization of symptoms due to frequent exposure [23–25]. Asymptomicity leads to low treatment-seeking behavior in populations with no access to surveillance or testing, which increases both the length of time people in a population are infectious and the exposure probability for susceptible people.

**Fig 1:**
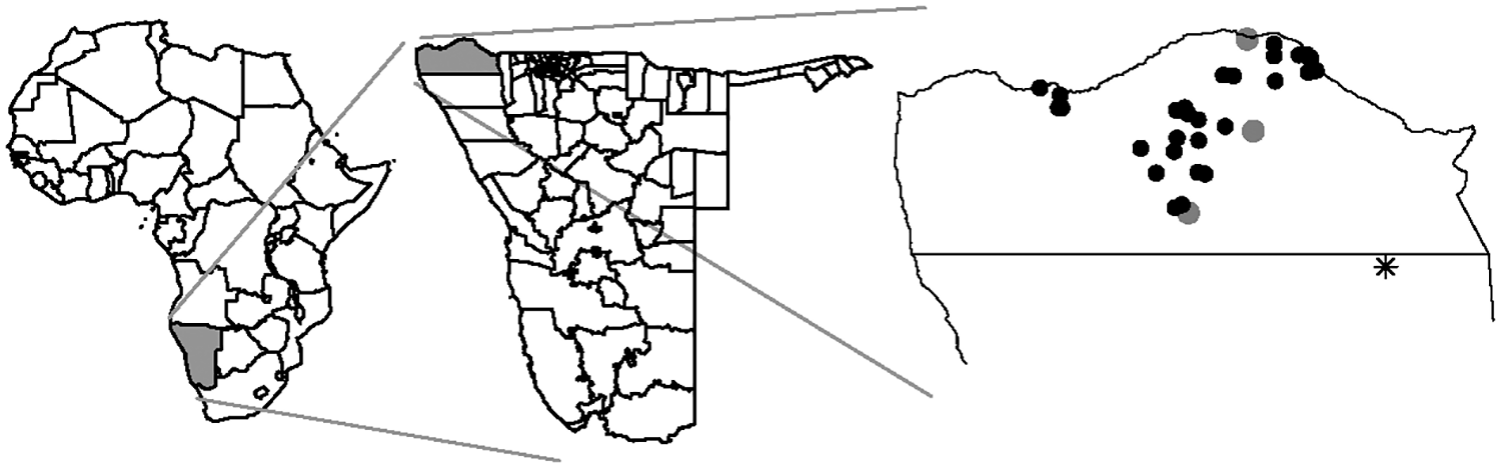
Map of Kaokoveld, Namibia: Black dots indicate location of 28 data-collection sites. Grey dots indicate locations of the four urban settlements in Kaokoveld. (Reprinted from Hazel, Foxman, & Low, 2015).

We have been conducting empirical research into the exposure and risk characteristics of HSV-2 among the semi-nomadic agro-pastoralist tribes of Kaokoveld in northwestern Namibia (Fig 1). In a previous study of HSV-2 prevalence in Kaokoveld [26], we found that, in addition to having a high overall prevalence (35%, women = 56%, men = 29%), HSV-2 infections were heterogeneously distributed geographically, with some regions having significantly higher prevalence than others.

While a high overall prevalence was not a surprising finding, given the cultural acceptability of concurrency (Table 1), significant geographic heterogeneity in prevalence was unexpected because pastoralist mobility coupled with very high prevalence would be expected to result in widespread transmission across the community in a way that would reduce heterogeneity. The variance in prevalence was not explained by urban proximity; in fact, qualitatively, it appeared that the regions with the highest HSV-2 prevalence were actually far away from urban areas or other regions [26].

**Table 1:** Current partner numbers among 441* participants in Kaokoveld Namibia, 2009.

Number of partners in prior 6 months	n (%)
0	31 (7%)
1	202 (46%)
2	78 (18%)
3	46 (10%)
>3	84 (19%)
**Proportion >1**	**208 (47%)**

*Five participants did not provide data on current partner numbers and were excluded from these totals.

The reported contacts between men and women are imbalanced and all our field experience indicates that this was mostly due to women underreporting (see Hazel, Marino & Simon 2015 for explanation). In any case, concurrency is very common for both sexes in Kaokoveld.

We explore here whether these high prevalence regions are more functionally remote, which we define as having higher rates of local mobility and local partner selection than other regions. If so, do these functionally remote regions have sexual network structures (small, densely connected) that foster accelerated STI transmission? To test our hypothesis that there is an association between remoteness and HSV-2 risk in Kaokoveld, we ask the following questions:

1. At the individual level, is there a relationship between having a higher proportion of partners from one’s residential region (partner homophily) and having HSV-2-specific antibodies? Are people who have a higher proportion of geographically homophilous partners more likely to have HSV-2?
2. If there is a relationship between homophily and HSV-2 positivity at the individual level, does the relationship hold at the regional level? Are the overall reported sexual contacts by people of some regions more likely to be homophilous than others?
3. If we say a region is *functionally* remote, we are saying that some factor other than simple distance prevents residents of that region from accessing the landscape, resources, and people of other regions, and we should be able to see the effect of that in people’s travel patterns. Thus, we ask: are people from regions with higher degrees of partner homophily also more likely to travel to destinations (villages, cattleposts) within their own region (geographic homophily)?

## Methods

### Study population, recruitment, and sample

In 2009, we conducted eight months of field-based interviews and HSV-2 diagnostics in 28 villages in Kaokoveld, Namibia. Using a cross-sectional design, we recruited any sexually active, culturally recognized adult residing in Kaokoveld at the time of study. Because most villages in Kaokoveld have a small number of people in residence at any one time (<50 adults), we used convenience sampling and interviewed all present adults who were willing to participate (N = 446; 213 women, 233 men). Twenty-seven participants were removed from the analyses because they either had an inconclusive HSV-2 test (n = 25) or refused the HSV-2 test (n = 2); thus our sample size for analyses was 419 (200 women, 219 men). Although we aimed to have equal numbers of participants in each age category (≤25 years, 26–35 years, 36–45 years, ≥46 years), 68% and 60% of our female and male samples, respectively, was made up of participants in the younger two age categories. However, the age distributions of participants were not significantly different by data-collection location; thus, our region-based analyses are not biased by differences in the samples’ age structures. Because HSV-2 is neither stigmatized nor consistently understood as a viral STI by the study community, we have no reason to think that our sample is biased by people’s perception of their HSV-2 status.

### Demographic and residential data

All participants completed a survey that included questions about demographics, location of current residence, and current sexual partnerships. Because of the high mobility of Kaokoveld residents, people’s “home” village (where they have longstanding familial or marital ties) are not always where they most recently resided; however, we were able to triangulate participants’ answers about their home villages with data regarding recent travel and time spent in non-home villages to ensure we were recording each participants’ main current residence. Villages fall into one of several locally recognized and delineated regions and, for each home village, we recorded its corresponding region. Because there were so many home villages represented in our dataset (>100) relative to our participant sample size, and because sample size per village was highly variable (range: 1–65), we analyzed participant location at the region level. The majority of study participants (n = 391) named home villages in one of five local Kaokoveld regions—Marienfluss (n = 51), Ozosemo (n = 91), Omaanda (n = 104), Ehama (n = 84), and Omunjandu (n = 61). Twenty-eight participants either lived in outside regions (including Angola, Kaokoveld peripheries, or outside of Kaokoveld, n = 18) or in one of the four Kaokoveld urban settlements (Opuwo, Okanguati, Epupa, Etanga, n = 10).

### Sexual-partner data

We asked participants to tell us the names of all people with whom they had had vaginal intercourse in the past six months, each partner’s home village, the last time they had sex, and the name of their partner’s spouse (if applicable). Because concurrency and extramarital sexual partnerships are broadly culturally acceptable in Kaokoveld, participants were generally forthcoming about their recent sexual partners. We detected some underreporting from women, especially married women, but given the detail of questions about participants’ sex partners, it was difficult for participants to exaggerate their partner numbers.

### Remoteness and mobility data

All participants were asked to name the three places besides their own village that they travel to most often, and to tell us when, why, and how they usually travel there. We then organized those locations into the same categories we used for aggregating participant and partner home villages into regions.

### HSV-2 rapid antibody test

All participants provided a finger-stick blood sample for an HSV-2-specific antibody rapid test (BioKit, Lexington, MA, USA). Our methods for scoring participants’ results are described in a previous publication [26]. Following our 2009 fieldwork, two studies were published that reported low sensitivity of the BioKit test in East Africa [27, 28]. However, although our prevalence estimates may be lower than reality, the demographic and geographic prevalence patterns in our data are likely realistic because there is no evidence that our false negatives were not equally applied across the dataset.

All research activities were approved by the University of Michigan’s Institutional Review Board (HUM00025104) and the Namibian Ministry of Health and Social Services. In additional to institutional ethical approval, we sought approval from local chiefs at each data-collection village and every participant provided oral assent prior to participation. All data-collection activities were performed privately and participant information was kept confidential throughout all stages of the research.

### Log-linear models

We fit a series of log-linear models to test our predictions about the relationship between HSV-2 prevalence and variable sexual mixing patterns in Kaokoveld. Log-linear models allow us to explore the impact of partner-selection at a higher level of complexity than more commonly used preferred-mixing models. Although preferred mixing includes a term for in-group preference (i.e., the proportion of partners of people from group *i* who are also from group *i*), it also assumes that all other partners (from groups *j*) are selected proportionally [29, 30]. While we know that people in Kaokoveld are overwhelmingly more likely to select sex partners from nearby geographic areas (i.e., geographic partner homophily is high in all regions), we suspect that variability in both homophily and heterophily influence transmission dynamics of HSV-2. In contrast to preferred mixing, log-linear models allow us to estimate heterogeneity in selective mixing across subpopulations (e.g., different regions in Kaokoveld) from a mixing matrix (Table 2) based on participants’ sexual-behavior data.

**Table 2:** Matrix of sexual mixing by region in Kaokoveld Namibia, 2009.

**Participant region**	**Partner region**							
	Marienfluss	Ozosemo	Omaanda	Ehama	Omunjandu	Others	Cities	
Marienfluss	90	0	0	1	2	4	3	100
Ozosemo	1	159	21	0	0	32	10	223
Omaanda	2	10	230	17	3	38	23	323
Ehama	0	0	17	128	23	8	16	192
Omunjandu	4	3	8	15	109	7	13	159
Others	0	3	3	1	1	28	6	42
Cities	1	1	6	0	0	3	11	22
	98	176	285	162	138	120	82	1061

We also fit a log-linear model to test whether there were significant differences in in-region and out-region travel among participants from different regions. Regions with significantly higher proportions of in-region travel (travel homophily) could be viewed as “functionally remote” and differences in regional remoteness were compared with geographic partner homophily and HSV-2 prevalence.

All models and statistical analyses were built in R 3.4.0 [31].

## Results

A quick descriptive look at a matrix of partnerships by region tells us that homophily is strong all over Kaokoveld (Table 2), but varies between regions. How much does differential homophily account for the distribution of HSV-2 and is there an epidemiologically relevant relationship between sexual homophily and geographic remoteness?

### Is there a relationship between the proportion of an individual’s homophilous contacts and their likelihood of having been exposed to HSV-2?

Using a univariate logistic regression model, we found a mildly significant relationship between individuals’ proportion of homophilous sexual partners and having HSV-2 (OR = 1.8; 95% CI: 1.04–3.13; p = 0.04). This weakly significant result indicates that, while high degrees of homophilous partnering accounts for some of the HSV-2 risk in Kaokoveld, other factors may be important as well. Also, the drivers of homophily may not be uniform across Kaokoveld; geographic homophily sometimes indicate cultural, familial, or personal preference, while in other cases, homophily indicates constraints on partner access and availability. Kaokoveld is diverse in its terrain and ecological characteristics but, overall, Kaokoveld could be described as remote, with some areas being more traversable than others, so that people in some areas may be less restricted by geography than others. It is also worth noting that the average number of partners over a six-month period also varies by region (Table 3).

**Table 3:** Number of contacts by region in Kaokoveld Namibia, 2009.

Number of contacts	Marienfluss	Ozosemo	Omaanda	Ehama	Omunjandu	Other villages	Cities
0	1	7	5	3	4	2	1
1	26	51	38	42	24	6	4
2	12	17	28	17	9	3	3
3	6	11	11	10	7	2	0
4	4	5	11	3	5	2	1
5	2	2	6	3	6	2	0
6	1	1	4	3	1	0	0
7	0	2	3	2	2	1	0
8	0	0	0	0	1	0	1
9	0	0	2	1	0	0	0
10	0	0	1	1	1	0	0
11	0	2	0	0	0	0	0
13	0	0	1	0	0	0	0
15	0	0	1	0	0	0	0
20	0	1	0	0	0	0	0
23	0	0	1	0	0	0	0
No. participants/region	52	99	112	85	60	18	10
Total contacts/region	100	210	325	184	151	43	22
Average number of contacts	1.923	2.121	2.902	2.165	2.517	2.389	2.200

### At the regional level, do we see a relationship between degree of homophily and HSV-2 prevalence?

We ran a set of log-linear models and found that the model accounting for differential homophily (i.e., where homophily varies by region, according to the mixing matrix; Table 2) is the best fit (Table 4). The Marienfluss (e^5.951^ = 384.1), Ozosemo (e^3.693^ = 40.2), and Omunjandu (e^3.031^ = 20.7) have the highest proportions of geographic homophily in sexual-partner selection compared with ranges of the coefficient exponents from Omaanda, Ehama, other regions, and Kaokoveld cities, which range from 3.9–14.9. Among our five sampled regions, the Marienfluss, Ozosemo, and Omunjandu also have the highest HSV-2 prevalences.

**Table 4:** Log-linear results for sexual mixing by region in Kaokoveld Namibia, 2009.

Parameter		Main effects only	Uniform homophily	Differential homophily
Reference category main effects		2.685	1.50	1.392
Participants				
Ozosemo		-0.101*	0.105*	-1.277
Omaanda		0.701	0.903	0.616
Ehama		1.071	1.00	1.192
Omunjandu		0.551	0.709	0.682
Outer areas		0.362	0.544	0.410
Cities		-0.969	-1.416	-0.649
Partners				
Ozosemo		-0.360	-0.293	-1.568
Omaanda		0.225	-0.288	-0.633
Ehama		0.707	0.115*	0.740
Omunjandu		0.142*	-0.219	0.076*
Outer areas		-0.018*	-0.254	-0.142*
Cities		-0.158*	0.608	0.901
Interactions				
Uniform			2.890	
Differential	Marienfluss			5.951
	Ozosemo			3.693
	Omaanda			2.113
	Ehama			2.701
	Omunjandu			3.031
	Outer areas			1.688
	Cities			1.354
AIC		2072	428.5	338.5
df		36	35	29

*p>0.05, not statistically significant; Marienfluss is reference category.

### Are regions with greater sexual-partner homophily more likely to be functionally remote?

To answer this question, we first needed to determine whether there was meaningful variation in the functional remoteness of the Kaokoveld regions. We ran a set of log-linear models to assess variation in mobility between the regions (Table 5). We found that within-region mobility was common overall, but that geographic mixing varied significantly by region. The regions with the highest proportion of within-region mobility were the Marienfluss, Ozosemo, and Omunjandu, with the Marienfluss and Ozosemo having the highest degree of homophilous mobility.

**Table 5:** Log-linear results for mobility by region in Kaokoveld Namibia, 2009.

Parameter		Main effects only	Uniform homophily	Differential homophily
Reference category main effects		2.843	2.191	2.277
Residence region				
Ozosemo		-0.026*	0.162	-0.351
Omaanda		0.791	1.072	0.804
Ehama		0.881	1.062	0.972
Omunjandu		0.636	0.800	0.606
Outer areas		0.327	0.429	0.183
Cities		-0.920	-1.242	-1.066
Destination region				
Ozosemo		-0.505	-0.432	-0.871
Omaanda		-0.526	-0.862	-1.285
Ehama		-0.073*	-0.403	0.035*
Omunjandu		-0.1428	-0.341	-0.242
Outer areas		-0.099*	-0.126*	-0.104*
Cities		0.490	0.859	1.00
Interactions				
Uniform			1.772	
Differential	Marienfluss			3.087
	Ozosemo			2.547
	Omaanda			1.147
	Ehama			1.777
	Omunjandu			1.974
	Outer areas			1.120
	Cities			-0.803
AIC		1112.4	589.5	509.5
df		36	35	29

*p>0.05, not statistically significant; Marienfluss is reference category.

**Fig 2:**
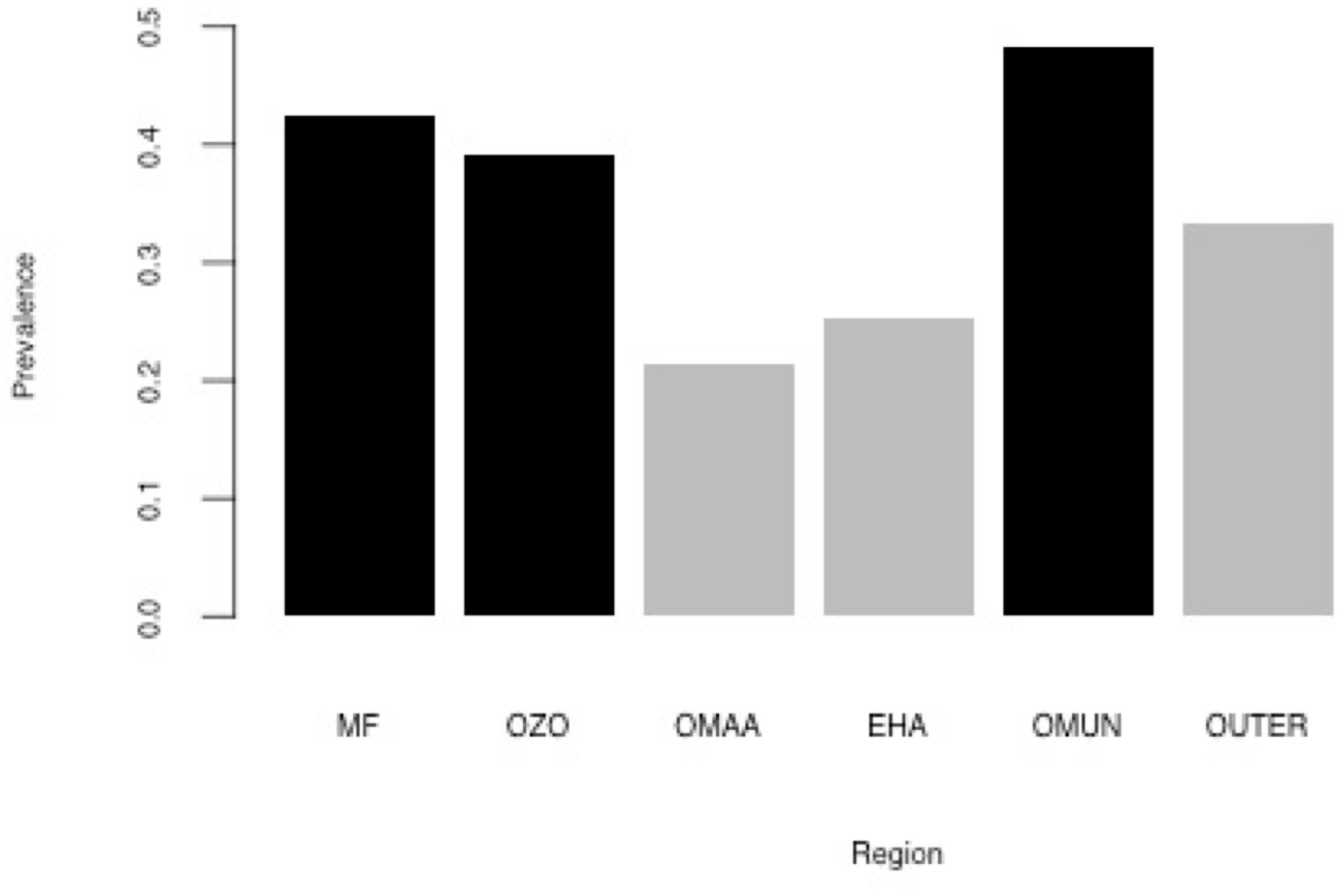
Herpes-simplex virus type-2 (HSV-2) prevalence by region. Black bars indicate regions with significantly higher prevalence than the reference category (Omaanda). MF = The Marienfluss; OZO = Ozosemo; OMAA = Omaanda; EHA = Ehama; OMUN = Omunjandu; OUTER = other regions and villages.

Given our assertion that higher proportions of within-region mobility indicates geographic isolation and limited access to outer areas, these three regions appear to be the most functionally remote in our dataset. The Marienfluss (42%), Ozosemo (39%), and Omunjandu (48%) are also the regions with the highest HSV-2 prevalences (Fig 2), further suggesting that remoteness is at least partially associated with high HSV-2 prevalence. In our sample, HSV-2 prevalence was 50% among participants who resided in urbanized towns of Kaokoveld (Opuwo, Okanguati, Epupa, Etanga), but the sample size was extremely small (n = 10) and unlikely to accurately reflect urban prevalence.

The Marienfluss is both remote and distant. By Euclidean distance, Marienfluss villages are ~170km from the region’s most common urban destination, Opuwo, which, despite being the farthest urban area from the Marienfluss, has the most traversable route throughout the year. Furthermore, the Marienfluss population is among the poorest in Kaokoveld, with none of the Marienfluss participants interviewed having a large enough herd to qualify as wealthy by local standards (>50 cows or >100 goats; our unpublished data from unstructured interviews with local informants in 2009). In fact, very few Marienfluss participants had any livestock at all (n = 8 out of 53). Thus, people in the Marienfluss have less money (e.g., to pay hitchhiking fees) and fewer reasons (e.g., to sell/trade cattle) to travel long distances. The relatively higher poverty in the Marienfluss could be exacerbating the effects of remoteness; thus, simultaneously increasing constraints on partner selection and further disadvantaging people from accessing treatment.

**Fig 3:**
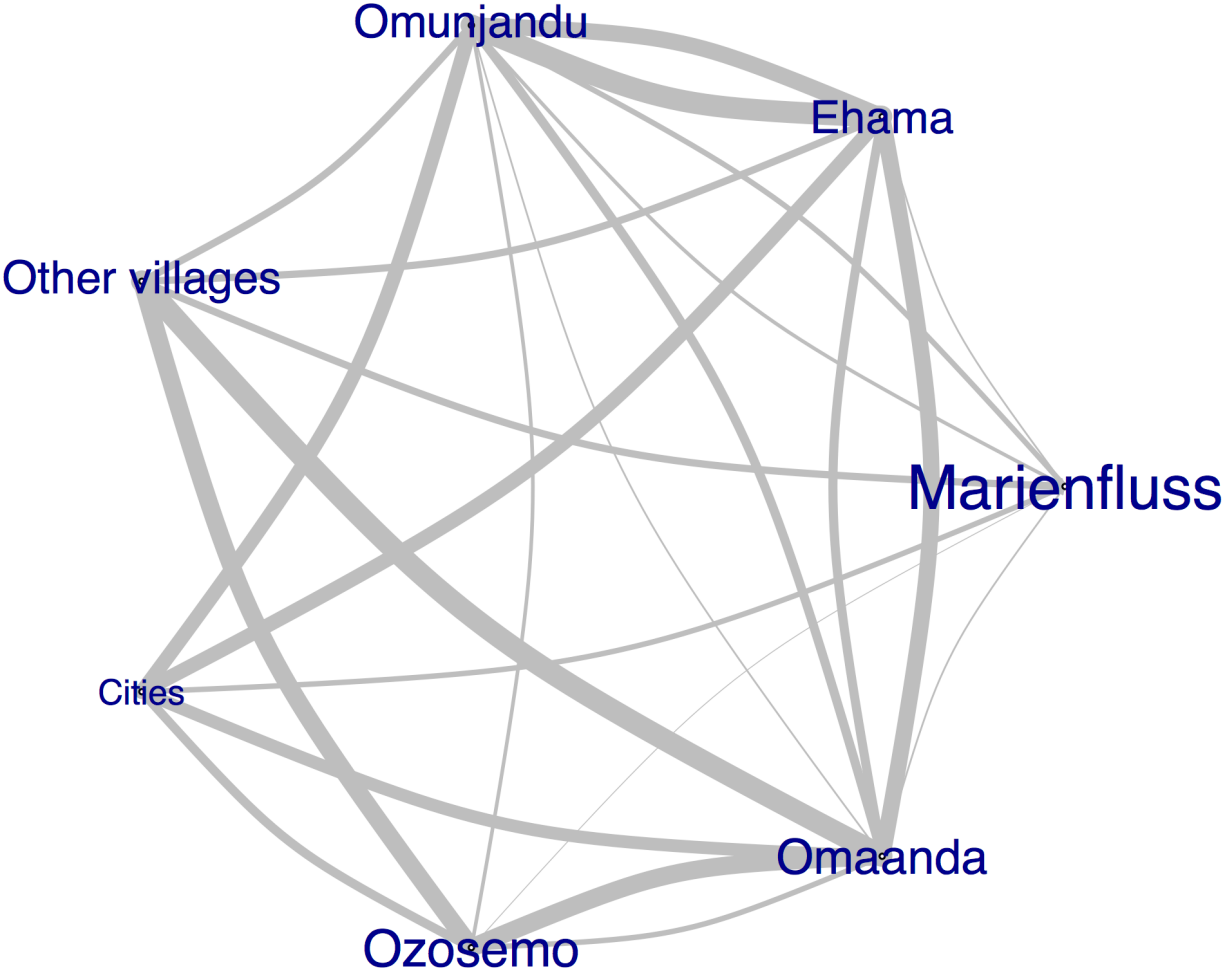
Mixing between regions: Region names are sized according to degree of homophily (bigger name indicates higher homophily) and the edges between the regions are sized according to amount of mixing between the regions (thicker edges indicate greater contact).

Compared with the Marienfluss, Ozosemo is more proximately located to its closest urban center (Epupa) and adjacent regions, but a gravel road that functions as the main artery for vehicle travel across Kaokoveld separates Ozosemo from the Marienfluss, Ehama, Omaanda, and Omunjandu. This fact does not functionally impede movement between the regions but may reflect a longstanding separateness. According to participant responses to our mobility questions, participants living in the Marienfluss, Ehama, Omaanda, and Omunjandu traveled to the urban areas of Okanguati, Opuwo, or Etanga for clinic care, livestock trading, and other economic or social purposes, while only Ozosemo residents primarily traveled to Epupa for these purposes. Partner homophily in Ozosemo was lower than in the Marienfluss (0.71 and 0.9, respectively), but Ozosemo and the Marienfluss were similar in that, compared to the other regions, they drew non-homophilous partners from the smallest range of external areas (Fig 3). Ozosemo is not geographically far, but mobility and partner selection are nevertheless constrained. Additionally, Ozosemo had the second lowest percentage of wealthy herd owners in our dataset, after the Marienfluss. Thus, further work in this area should explore the relationship between poverty and remoteness, even within seemingly homogenous communities.

Omunjandu is more centrally located than either the Marienfluss or Ozosemo, and actually sits between Omaanda and Ehama. And, although Omunjandu had the lowest degree of within-region mobility and sexual-partner homophily of the three remote regions, it also had the highest HSV-2 prevalence (48%). Furthermore, Omunjandu’s coefficients for differential within-region mobility and homophily were similar, though larger, than Ehama’s. While our log-linear results for differential mobility and homophily were significant for all regions, including Ehama and Omunjandu, the stark differences in HSV-2 prevalence between the regions may be due to an insignificant difference in the age structure of the samples we collected in Ehama and Omunjandu. Our sample in Ehama contained a larger number of members of the youngest age group (≤25 years old) than Omunjandu, which contained a larger number of members of the oldest age group (≥45 years old). Consistent with most epidemiologic investigations of HSV-2 prevalence [32–36], we identified a strong association between HSV-2 and older age in Kaokoveld [26]. Although our data indicate that older people in Kaokoveld travel less (participants ≤35 years old reported 823 total trips while those ≥35 years old reported 458 total trips), they are not more likely to restrict their travel to local destinations. Furthermore, although the age-group difference between regions in our sample was not significant (*X*^*2*^ = 19.6, df = 18, p = 0.36), it probably skewed the prevalence differences between the regions to be artificially greater than reality, suggesting that Omunjandu may not be as functionally remote nor as highly risky for HSV-2 exposure as our data suggest, although a sub-sample biased toward older participants does not completely explain the behavioral and epidemiological patterns in Omunjandu.

Thus far, we have discussed how our empirical work in Kaokoveld helps us understand very localized transmission dynamics of HSV-2, an endemic, largely low-morbidity viral STI. However, even highly remote rural communities like those of the Kaokoveld pastoralists are not social or epidemiologic islands. There is mobility in and out of Kaokoveld from other parts of rural Namibia and Angola, with growing evidence that rural-urban mobility is becoming increasingly common [37] (Hazel, unpublished). More movement between rural and urban areas in Kaokoveld may be slow to increase sexual contact between members of the pastoralist community and non-members because cultural taboos curtail sex with outsiders [26, 38], but increased exposure and changing ecology and social mores may eventually erode those taboos. More immediately, however, these emerging mobility patterns could foster more contact between heterophilous community members because increased urban movement, made possible by vehicular travel, could increase sexual-partnership formation between rural-living people when visiting urban locales.

We argue that heterophilous bridging is particularly epidemiologically significant when it involves densely connected small networks, such as in Kaokoveld, which allow for accelerated disease transmission on a local scale. Increasing contact bridges between dense networks may result in locally amplified viral transmission that can pulse out (or back in) on a broader geographic scale, especially when bridges form with populations with high proportions of susceptible people. Rural communities undergoing ecological or subsistence changes, particularly in ways that affect mobility and social contact structures, are important to this dynamic: they can be the source of epidemic acceleration and importation to newly contacted communities or they can be the recipients of newly imported pathogens, which, upon entry into small highly connected sexual networks, can spread rapidly.

**Fig 4:**
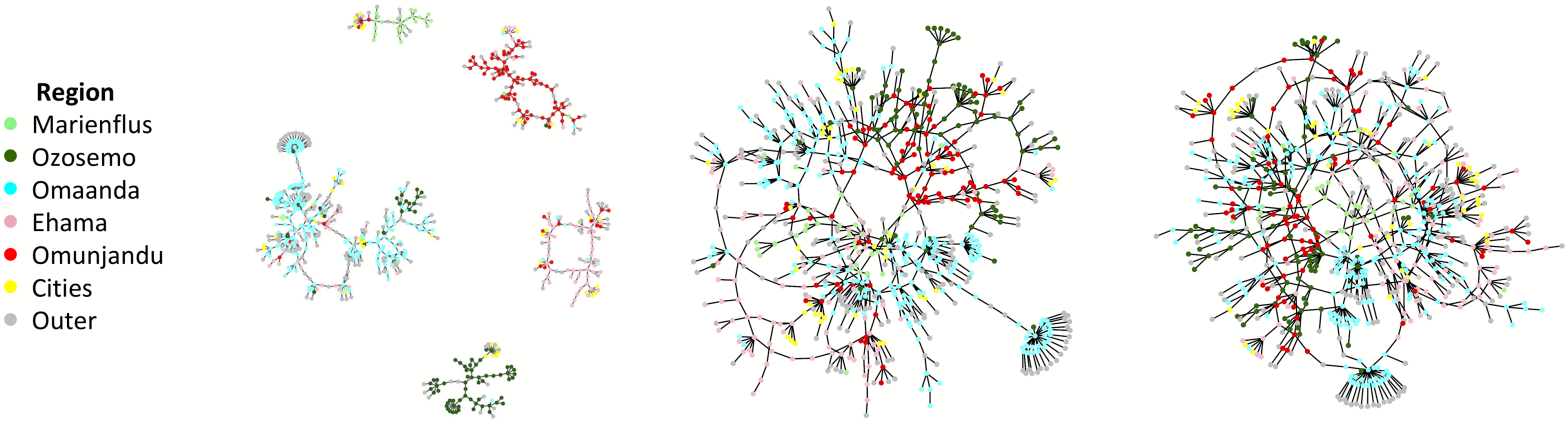
**a-c**. Network graphs of three hypothetical models: 4a) Hypothetical network constructed from structural elements of Kaokoveld network representing the five main regions and their mixing dynamics. Structures were simplified so that no regional subcomponents had any ties between them at T_0_. 4b) Hypothetical network from (4a) with 3% more ties between the subcomponents, all added at random. 4c) Hypothetical network from (4b) with 2% more ties (5% more than (4a)) between the subcomponents, all added at random.

To demonstrate the epidemiological effects of bridging in highly homophilous networks, we simulated a simple viral STI epidemic on a set of hypothetical heterosexual network models with structural and mixing features mimicking those in our Kaokoveld dataset. The graph in Fig 4a is composed of five distinct network subcomponents that approximately represent the differential homophily and heterophily of the five main Kaokoveld regions. The subcomponents all include heterophilous contacts but no connections are permitted between the subcomponents at the initial T = 0. Fig 4b and 4c show the same structures as Fig 4a, except we rewired them to include 3% and 5% more random ties, respectively, across the subcomponents. Although preference for ties between homophilous nodes in Fig 4b & 4c are clearly still dominant, even modest amounts of bridging can erode boundaries between subcomponents.

**Fig 5:**
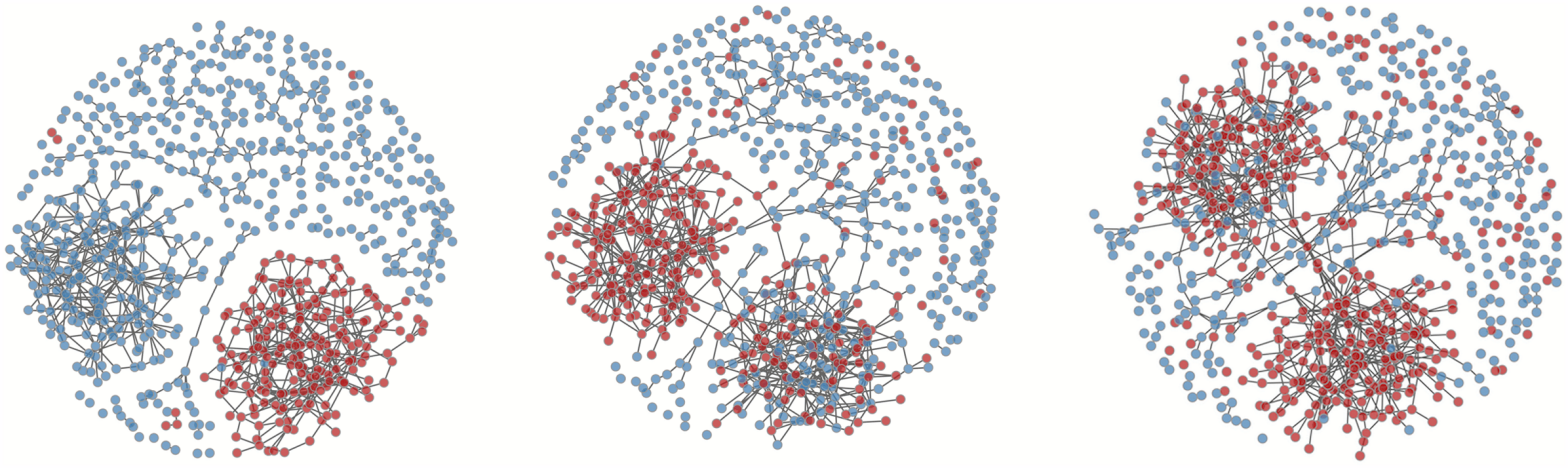
**a-c**. Network graphs of simulations on graphs from Fig 4a–4c after transmission of an HSV-2-like virus at T_250_ (~8 months), blue nodes = susceptible; red nodes = infectious: 5a) Simulation on network with zero ties at T_0_ (Fig 4a); 5b) Simulation on network with 3% more ties between subcomponents at T_0_ (Fig 4b); Simulation on network with 5% more ties between subcomponents at T_0_ (Fig 4c).

When we simulated these networks to form and dissolve ties over time with a very simplistic HSV-2-like epidemic dynamic running over the tie-formation and tie-dissolution models, the virus spread faster and reached more elements of the whole network as bridging across subcomponents increased (Fig 5a–5c). In the least connected network (Figs 4a and 5a), the virus spreads completely throughout a single subcomponent but the nodes in all the other structures of the network mostly remain susceptible. As we increased connectivity, the virus spread faster and further—overwhelming another giant subcomponent (Fig 5b) before spreading into other smaller structures through the network (Fig 5c).

**Fig 6:**
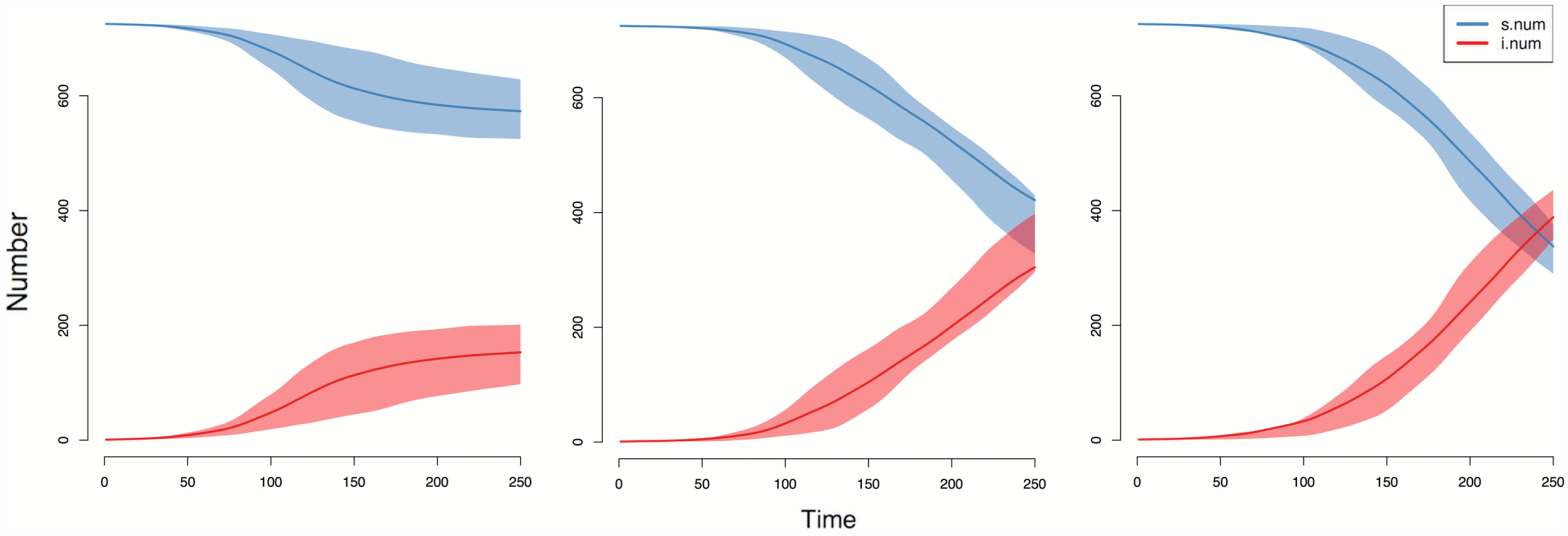
**a-c**. Infection dynamics for three hypothetical epidemic simulations (Fig 5a–5c): 6a) Number of infected individuals increases but the infection rate slowed after ~T_150_. Susceptibles still dominate the population. 6b) Number of individuals increases and, in contrast to (6a), the infection rate increases after T_150_. 6c) The infection rate increases similarly but at a faster rate than (6b) and by T_250_, the number of infected individuals has surpassed susceptibles.

Robust models of HSV-2 dynamics account for the virus’s dormancy periods and variable shedding rates, and they also consider other host biological characteristics (e.g., prevalence of HSV-1, partner sero-discordance rate), all of which prevent real-world endemic HSV-2 from completely saturating a population [39]. However, since we lack necessary data from Kaokoveld for more precise HSV-2 modeling, we modeled a simple viral STI that has very low per-act transmissibility (1.2%) but also has lifelong infectiousness (i.e. a susceptible-infected, or SI, model). Given enough time, simple SI models always reach 100% prevalence, so we focus, not on the equilibrium points, but on the progression of the epidemic after 250 time steps (~eight months; approximately the earliest time step at which equilibrium is approached in the three models). The epidemic moves faster with increasing subcomponent bridging (Fig 6a–6c) and the number of infected nodes surpasses the number of susceptible nodes in our simulations of the model with 5% subcomponent bridging.

## Discussion

HSV-2 is more prevalent in Kaokoveld regions that are functionally remote, which is to say, regions where travel and selection of sexual partners is biased toward proximate targets. When coupled with a culturally normalized practice of partner concurrency, remoteness facilitates the formation of small, densely connected sexual networks, which are highly effective at spreading viral STIs.

Carnegie & Morris [40] showed how densely connected networks can emerge in small populations with low average lifetime partners if even modest amounts of partner concurrency are common, as in many sub-Saharan African contexts. Because rural populations are often small, deep connectivity can emerge with even low-level concurrency (2–3 partners) and low lifetime partner numbers. According to the sexual partner data we collected from our participants, 47% had more than one current partner (as defined as having had sex within the past six months); 19% had more than three partners (Table 1).

In remote communities with high HSV-2 prevalence, small population size, densely connected sex networks, and low access to potential partners in other networks, people who are selecting new partners are more likely to select someone who is seropositive than someone who is selecting from a potentially larger pool. Even with higher contact rates (e.g., Omaanda (2.9) vs. Marienfluss (1.9), Table 3), having a more heterophilous partner pool may increase the likelihood of selecting a seronegative partner. In fact, the average number of current partners (over the previous six months) was lowest among participants from the Marienfluss and Ozosemo, the two regions with the highest partner homophily. Thus, even though the individual-level behavior of people in the high-prevalence regions was less risky, because of the effects of remoteness (more homophilous mixing, smaller and denser sexual networks), people living in these regions were embedded in riskier networks.

In circumstances with modest partner concurrency, having fewer concurrent partners may increase the per-act contact rate for a given partnership, which, when one of those partners is seropositive, would increase the likelihood of HSV-2 transmission between that partner set. The HSV-2 per-act transmission rate is very low (<3% [39]), so its transmission dynamics are usually modeled using a per-partnership rate [41].

What we are observing in Kaokoveld is that, one’s network, as opposed to their personal behavior, is the primary source of relative viral STI risk, which is a structural type of risk that is not unique to Kaokoveld or even remote subsistence-living people. In Kaokoveld, where concurrency is culturally acceptable and broadly practiced, remoteness constrains partner choice. We can compare the geographic constraints in Kaokoveld to different constraining phenomena in the United States where African-American women have a statistically-indistinguishable number of sexual partners from white women and are often less likely to be involved in risky sexual behavior than white women, yet they contract STIs at much higher rates. This is because, despite the fact that the behavior among African-American women is no more risky than that of other American women, they are more likely to have partners with riskier behavior [42–44]. The risky networks in which African-American women are embedded are analogous to those in Kaokoveld; however, in contrast to remote-living women in Kaokoveld, African-American women are not constrained by landscape but, rather, they experience greater social, economic, and institutional remoteness than other American women [45], which amplifies risk in their networks [46]. Making the distinction between network-embedded risk and individual- behavioral risk is critical because behavioral-focused interventions are unlikely to be effective against the more complex dynamics underlying structural risk.

Our network simulations show that, when coupled with strong in-group preferences, very small increases in out-group contact can have powerful impacts on epidemic dynamics in small remote populations. Despite the fact that travel is still mostly rural in Kaokoveld, access to urban areas has increased with greater presence of automobiles. Furthermore, urban populations are not only growing but are increasingly populated by people settling from outside Kaokoveld. Population growth for Opuwo, the capital city of the Kunene district and the largest urban center of Kaokoveld, was over 4% between 2001–2011, which is greater than the growth in Namibia’s two largest cities (Windhoek, Oshakati) and twice as much as growth for the entire Kunene district [47]. Kaokoveld is within the Kunene province, which has one of the lowest HIV prevalences in Namibia, but the other provinces along Namibia’s northern boarder, Oshana, Ohangwena, and Omusati, have among the highest HIV prevalences and some of the densest populations in Namibia [48]. These changing population and mobility dynamics might not break up the clusters in the Kaokoveld networks, especially in highly remote and homophilous areas, but initial mobility changes may lead to more bridges into those networks, increasing the likelihood of HIV importation or the spread of more virulent strains of endemic diseases (e.g., resistant gonorrhea).

We see relevant parallels between the network structures underlying HIV spread across sub-Saharan Africa and present structures in Kaokoveld which are subject to change in response to rapidly changing economic, social, and ecological environments. The HIV pandemic that swept across eastern [49–51] and southern Africa [52] spread through major labor migration routes (e.g., long-haul truckers and miners) and amplified in the small local networks of rural settlements. Among some of the first studies that investigated the transmission pathways of HIV in sub-Saharan Africa, Bwayo et al. [49] identified long-haul truckers in Kenya as a high-risk or “core” group, and that, among truckers surveyed in Kenya, seroprevalence increased with distance traveled. Extremely high HIV prevalence was also common among female sex-workers (e.g., 76% seroprevalence among barmaids in Uganda) who serviced long-haul truckers in East Africa [53], but these women had very local mobility patterns [54], indicating that sex-worker communities served as bridges to their local communities and small networks, while truckers and migrant workers remained incorporated in their hometown and villages networks. Among high-risk groups in South Africa, Ramjee et al. [52] found that the female partners of migrant workers, including long-haul truckers, were more likely than the partners of non-migrant workers to take additional partners when their spouses were gone, leading to increased density of small local networks and accelerated local HIV spread.

The type of commerce and mobility that fostered HIV spread across broad regions of sub-Saharan Africa are not present in northwestern Namibia or many other remote rural areas of Africa. However, climate-change-induced alterations to local ecologies, in conjunction with increased urbanization, may have big impacts on rural livelihoods, especially for subsistence-living people. We warn that, along with alterations to transmission pathways that increase exposure, remote communities could see importation of novel STIs, and their densely connected networks of high proportions of susceptible people would foster rapid epidemic spread. Current HSV-2 transmission trends may serve as a bellwether for potential HIV spread into Kaokoveld from the high HIV prevalence and densely populated adjacent districts. There is also evidence that, in rural sub-Saharan African communities with high HIV prevalence, ecological shocks (e.g., decreased rainfall) drive household economic decisions that lead to increased HIV transmission [55]. Drought intensification will force ecological and economic adaptations for many rural living Africans; we cannot ignore the fact that such adaptations can dramatically alter sexual and support networks in ways that increase STI exposure—including but not limited to HSV-2—among communities who already struggle for lack of access to preventive care and treatment.

## References

[1] Bhutta ZA, Sommerfeld J, Lassi ZS, Salam RA, Das JK. Global burden, distribution, and interventions for infectious diseases of poverty. Infectious Diseases of Poverty. 2014;3(21):1–7. doi: 10.1186/2049-9957-3-21 25110585 10.1186/2049-9957-3-21 PMC4126350

[2] Bonds MH, Keenan DC, Rohani P, Sachs JD. Poverty trap formed by the ecology of infectious diseases. Proceedings of the Royal Society of London B: Biological Sciences. 2010;277(1685):1185–92. doi: 10.1098/rspb.2009.1778 20007179 10.1098/rspb.2009.1778 PMC2842808

[3] Lui L, Johnson HL, Cousens S, Perin J, Scott S, Lawn JE, et al Global, regional, and national causes of child mortality: An updated systematic analysis for 2010 with time trends since 2000. Lancet. 2012;379(June 9):2151–61. 22579125 10.1016/S0140-6736(12)60560-1

[4] Gottlieb SL, Low N, Newman LM, Bolan G, Kamb M, Broutet N. Toward global prevention of sexually transmitted infections (STIs): The need for STI vaccines. Vaccine. 2014;32(14):1527–35. doi: 10.1016/j.vaccine.2013.07.087 . 24581979 10.1016/j.vaccine.2013.07.087 PMC6794147

[5] Singh S, Darroch JE, Ashford LS. Adding it up: The costs and benefits of investing in sexual and reproductive health 2014. Guttmacher Institute, 2014.

[6] The World Health Organization. Global prevalence and incidence of selected curable sexually transmitted infections: Overview and estimates. Geneva, Switzerland: WHO, 2001.

[7] Kim JY, Farmer PE, Porter ME. Redefining global health-care delivery. The Lancet. 2013;382(9897):1060–9. doi: 10.1016/S0140-6736(13)61047-8 10.1016/S0140-6736(13)61047-8 23697823

[8] Strasser R. Rural health around the world: Challenges and solutions. Family Practice. 2003;20(4):457–63. doi: 10.1093/fampra/cmg422 12876121 10.1093/fampra/cmg422

[9] Tanser FC, Gijsbertsen B, Herbst K. Modelling and understanding primary health care accessibility and utilization in rural South Africa: An exploration using a geographical information system. Social Science & Medicine. 2006;63:691–705. doi: 10.1016/j.socscimed.2006.01.015 16574290 10.1016/j.socscimed.2006.01.015

[10] Tanser FC. Methodology for optimising location of new primary health care facilities in rural communities: A case study in KwaZulu-Natal, South Africa. Journal of Epidemiology and Community Health. 2006;60(10):846–50. doi: 10.1136/jech.2005.043265 16973529 10.1136/jech.2005.043265 PMC2566049

[11] Linard C, Gilbert M, Snow RW, Noor AM, Tatem AJ. Population distribution, settlement patterns and accessibility across Africa in 2010. PLoS One. 2012;7(2):e31743 doi: 10.1371/journal.pone.0031743 . 22363717 10.1371/journal.pone.0031743 PMC3283664

[12] Zinsstag J, Taleb MO, Craig PS. Health of nomadic pastoralists: New approaches towards equity effectiveness. Tropical Medicine and International Health. 2006;11(5):565–8. doi: 10.1111/j.1365-3156.2006.01615.x 16640607 10.1111/j.1365-3156.2006.01615.x

[13] Wakerman J. Defining remote health. Australian Journal of Rural Health. 2004;12:210–4. doi: 10.1111/j.1440-1854.2004.00607.x 15588265 10.1111/j.1440-1854.2004.00607.x

[14] Bharti N, Tatem AJ, Ferrari MJ, Grais RF, Djibo A, Grenfell BT. Explaining seasonal fluctuations of measles in Niger using nighttime lights imagery. Science. 2011;334(6061):1424–7. doi: 10.1126/science.1210554 . 22158822 10.1126/science.1210554 PMC3891598

[15] Wesolowski A, Eagle N, Tatem AJ, Smith DL, Noor AM, Snow RW, et al Quantifying the impact of human mobility on malaria. Science. 2012;338(6104):267–70. doi: 10.1126/science.1223467 . 23066082 10.1126/science.1223467 PMC3675794

[16] Funk S, Salathé M, Jansen AA. Modelling the influence of human behaviour on the spread of infectious diseases: A review. Journal of The Royal Society Interface. 2010:1–10. doi: 10.1098/rsif.2010.0142 20504800 10.1098/rsif.2010.0142 PMC2894894

[17] Borgerhoff Mulder M, Bowles S, Hertz T, Bell A, Beise J, Clark G, et al Intergenerational wealth transmission and the dynamics of inequality in small-scale societies. Science. 2009;326(October 30):682–8. doi: 10.1126/science.1178336 19900925 10.1126/science.1178336 PMC2792081

[18] Low BS. Women’s lives there, here, then, now: a review of women’s ecological and demographic constraints cross-culturally. Evolution and Human Behavior. 2005;26(1):64–87. doi: 10.1016/j.evolhumbehav.2004.08.011

[19] Scelza BA. Choosy But Not Chaste: Multiple Mating in Human Females. Evolutionary Anthropology: Issues, News, and Reviews. 2013;22(5):259–69. doi: 10.1002/evan.21373 24166926 10.1002/evan.21373

[20] Pennington R, Harpending H. The Structure of an African Pastoralist Community: Demography, History, and Ecology of the Ngamiland Herero. Oxford, UK: Clarendon Press; 1993 268 p.

[21] Hudson CP. Concurrent Partnerships could cause AIDS epidemics. International Journal of STD & AIDS. 1993;4:249–53. doi: 10.1177/095646249300400501 8218510 10.1177/095646249300400501

[22] Morris M, Kretzschmar M. Concurrent partnerships and the spread of HIV. AIDS. 1997;11(5):641–8. 9108946 10.1097/00002030-199705000-00012

[23] Wilkinson D, Abdool Karim SS, Harrison A, Lurie M, Colvin M, Connolly C, et al Unrecognized sexually transmitted infections in rural South African women: A hidden epidemic. Bulletin of the World Health Organization. 1999;77(1):22–8. 10063657 PMC2557569

[24] Plummer FA, Simonsen JN, Chubb H, Slaney L, Kimata J, Bosire M, et al Epidemiological evidence for the development of serovar-specific immunity after gonoccocal infection. Journal of Clinical Investigation. 1989;83(5):1472–6. doi: 10.1172/JCI114040 2496142 10.1172/JCI114040 PMC303849

[25] Detels R, Green AM, Klausner JD, Katzenstein D, Gaydos C, Handsfield HH, et al The Incidence and Correlates of Symptomatic and Asymptomatic Chlamydia Trachomatis and Neisseria Gonorrhoeae Infections in Selected Populations in Five Countries. Sexually Transmitted Diseases. 2011:1. 22256336 PMC3408314

[26] Hazel A, Foxman B, Low BS. Herpes simplex virus type 2 among mobile pastoralists in northwestern Namibia. Annals of Human Biology. 2015;42(6):543–51. doi: 10.3109/03014460.2014.970575 25387244 10.3109/03014460.2014.970575

[27] Lingappa J, Nakku-Joloba E, Magaret A, Friedrich D, Dragavon J, Kambugu F, et al Sensitivity and specificity of herpes simplex virus-2 serological assays among HIV-infected and uninfected urban Ugandans. Int J STD AIDS. 2010;21(9):611–6. doi: 10.1258/ijsa.2009.008477 . 21097732 10.1258/ijsa.2009.008477 PMC3057112

[28] Ng’ayo MO, Friedrich D, Holmes KK, Bukusi E, Morrow RA. Performance of HSV-2 type specific serological tests in men in Kenya. J Virol Methods. 2010;163(2):276–81. doi: 10.1016/j.jviromet.2009.10.009 . 19854222 10.1016/j.jviromet.2009.10.009 PMC2820239

[29] Jacquez JA, Simon CP, Koopman J, Sattenspiel L, Perry T. Modeling and analyzing HIV transmission: The effect of contact patterns. Math Biosci. 1988;92:119–99. doi: 10.1016/0025-5564(88)90031-4

[30] Simon CP, Jacquez JA. Reproduction numbers and the stability of equilibria of SI models for heterogeneous populations. SIAM Journal of Applied Mathematics. 1992;52(2):541–76. doi: 10.1137/0152030

[31] The R Core Team. R 3.4.0. Vienna, Austria: R Foundation for Statistical Computing; 2017.

[32] Looker KJ, Garnett GP, Schmid GP. An estimate of the global prevalence and incidence of herpes simplex virus type 2 infection. Bulletin of the World Health Organization. 2008;86:805–12A. doi: 10.2471/BLT.07.046128 18949218 10.2471/BLT.07.046128 PMC2649511

[33] Mertz GJ. Epidemiology of genital herpes infections. Infect Dis Clin North Am. 1993;7(4):825–39. . 8106731

[34] Wald A. Herpes simplex virus type 2 transmission: Risk factors and virus shedding. Herpes. 2004;11 Suppl 3:130A–7A. . 15319082

[35] Watson-Jones D, Weiss HA, Rusizoka M, Baisley K, Mugeye K, Changalucha J, et al Risk factors for herpes simplex virus type 2 and HIV among women at high risk in northwestern Tanzania: Preparing for an HSV-2 trial. Journal of Acquired Immune Deficiency Syndrome. 2007;46(5):631–42. 10.1097/QAI.0b013e31815b2d9c PMC2643092 18043318

[36] Kirakoya-Samadoulougou F, Nagot N, Defer MC, Yaro S, Fao P, Ilboudo F, et al Epidemiology of herpes simplex virus type 2 infection in rural and urban Burkina Faso. Sex Transm Dis. 2011;38(2):117–23. 20838363 10.1097/OLQ.0b013e3181f0bef7

[37] Cameron AS. The Influence of Media on Himba Conceptions of Dress, Ancestral and Cattle Worship, and the Implications for Culture Change: Brigham Young University; 2013.

[38] Talavera P. Challenging the Namibian Perception of Sexuality: A Case Study of the Ovahimba and Ovaherero Culturo-Sexual Models in Kunene North in an HIV/AIDS Context. Windhoek, Namibia: Gamsberg, Macmillan; 2002 111 p.

[39] Schiffer JT, Mayer BT, Fong Y, Swan DA, Wald A. Herpes simplex virus-2 transmission probability estimates based on quantity of viral shedding. J R Soc Interface. 2014;11(95):20140160 doi: 10.1098/rsif.2014.0160 . 24671939 10.1098/rsif.2014.0160 PMC4006256

[40] Carnegie NB, Morris M. Size matters: Concurrency and the epidemic potential of HIV in small networks. PLoS ONE. 2012;7(8):1–6. 22937011 10.1371/journal.pone.0043048 PMC3427300

[41] Abu-Raddad LJ, Magaret AS, Celum C, Wald A, Longini IM Jr., Self SG, et al Genital herpes has played a more important role than any other sexually transmitted infection in driving HIV prevalence in Africa. PLoS One. 2008;3(5):e2230 doi: 10.1371/journal.pone.0002230 . 18493617 10.1371/journal.pone.0002230 PMC2377333

[42] Cooper HLF, Linton S, Haley DF, Kelley ME, Dauria EF, Karnes CC, et al Changes in exposure to neighborhhod characteristics are associated with sexual network characteristics in a cohort of adults relocating from public housing. AIDS Behavior. 2015;19:1016–30. doi: 10.1007/s10461-014-0883-z 25150728 10.1007/s10461-014-0883-z PMC4339671

[43] Finer LB, Darroch JE, Singh S. Sexual partnership patterns as a behavioral risk factor for sexually transmitted diseases. Family Planning Perspectives. 1999;31(5):228–36. 10723647

[44] Laumann EO, Youm Y. Racial/ethnic group differences in the prevalence of sexually transmitted diseases in the united states: A network explanation. Sexually Transmitted Diseases. 1999;26(5):250–61. 10333277 10.1097/00007435-199905000-00003

[45] Adimora A, Schoenbach VJ, Bonas DM, Martinson FEA, Donaldson KH, Stancil TR. Concurrent sexual partnerships among women in the United States. Epidemiology. 2002;13(3):320–7. doi: 10.1097/00001648-200205000-00013 11964934 10.1097/00001648-200205000-00013

[46] Morris M. A log-linear modeling framework for selective mizing. Math Biosci. 1991;107(2):349–77. 1806123 10.1016/0025-5564(91)90014-a

[47] Namibia Statistics Agency. 2001, 2011, 2016. https://www.citypopulation.de/Namibia.html. [cited June 19, 2017].

[48] The Namibian Ministry of Health and Social Services (MoHSS), International ICF. The Namibia Demographic and Health Survey 2013. Windhoek, Namibia and Rockville, MD, USA: MoHSS and ICT International; 2014.

[49] Bwayo J, Plummer F, Omari M, Mutere A, Moses S, Ndinya-Achola J, et al Human immunoficiency virus infection in long-distance truck drivers in East Africa. Archives of Internal Medicine. 1994;154:1391–6. doi: 10.1001/archinte.1994.00420120123013 8002691

[50] Bwayo JJ, Mutere AN, Omari MA, Kreiss JK, Jaoko W, Sekkade-Kigondu C, et al Long distance truck drivers: 2. Knowledge and attitudes concerning sexually transmitted diseases and sexual behaviour. East Afr Med J. 1991;68(9):714–9. . 1797534

[51] Bwayo JJ, Omari AM, Mutere AN, Jaoko W, Sekkade-Kigondu C, Kreiss J, et al Long distance truck-drivers: 1. Prevalence of sexually transmitted diseases (STDs). East Afr Med J. 1991;68(6):425–9. . 1752221

[52] Ramjee G, Gouws E. Prevalence of HIV among truck drivers visiting sex workers in KwaZulu-Natal, South Africa. AIDS and Behavior. 2002;15(4):687–92. 10.1097/00007435-200201000-00008 11773878

[53] Kreiss JK, Korech D, Plummer FA, Holmes KK, Lightfoote M, Piot P, et al AIDS virus infection in Nairobi prostitutes. The New England Journal of Medicine. 1986;314(7):414–8. doi: 10.1056/NEJM198602133140704 3484804 10.1056/NEJM198602133140704

[54] Carswell JW, Lloyd G, Howells J. Prevalence of HIV-1 in East African lorry drivers. AIDS. 1989;3:759–61. doi: 10.1097/00002030-198911000-00013 2515882 10.1097/00002030-198911000-00013

[55] Burke M, Gong E, Jones K. Income Shocks and HIV in Africa. The Economic Journal. 2014;125(June):1157–89. doi: 10.1111/ecoj.12149

